# Surface Hydrophobicity and Functional Properties of Citric Acid Cross-Linked Whey Protein Isolate: The Impact of pH and Concentration of Citric Acid

**DOI:** 10.3390/molecules23092383

**Published:** 2018-09-18

**Authors:** Tong Li, Chunyan Wang, Tianqi Li, Ling Ma, Dongxue Sun, Juncai Hou, Zhanmei Jiang

**Affiliations:** Key Laboratory of Dairy Science (Northeast Agricultural University), Ministry of Education, Harbin 150030, China; 18846939169@163.com (T.L.); 15504600693@163.com (C.W.); 15754501482@163.com (T.L.); 18846792903@163.com (L.M.); 13244601808@163.com (D.S.)

**Keywords:** whey protein isolate, citric acid, cross-link, surface hydrophobicity, functional properties

## Abstract

The effects of citric acid-mediated cross-linking under non-acidic conditions on the surface hydrophobicity, solubility, emulsifying, and foaming properties of whey protein isolate (WPI) were investigated. In this research, citric acid-mediated cross-linking could not only increase the surface hydrophobicity of whey proteins at pH 7.0 and 8.0, but it also improved its emulsifying and foaming properties. The emulsifying activity and foaming ability of WPI reached a maximum under the condition of 1% citric acid and pH 7.0. However, the solubility of WPI-CA gradually decreased with pH and the content of citric acid increased. Therefore, the cross-linking mediated by citric acid under non-acidic aqueous conditions, markedly altered the surface hydrophobicity and enhanced emulsifying and foaming properties of WPI.

## 1. Introduction

Whey protein is a general term for various protein components from whey. Different processes for protein extraction could produce whey protein products with different protein compositions, such as whey protein concentrate (WPC) and whey protein isolate (WPI) [1,2]. WPI has a high protein content, mainly including bovine serum albumin (BSA), β-lactoglobulin (β-Lg), and α-lactalbumin (α-La). WPI is an important protein ingredient and functional additive in the food industry, due to its good functional properties. Meanwhile, since whey protein molecules have free amino groups and carboxyl groups, it is easy to carry out covalent cross-linking in different ways [3]. In recent years, with the development of science and technology, there are an increasingly more research studies on the modification of protein. The methods of modification include enzyme cross-linking [4], chemical cross-linking [5], etc. WPI is suitably modified to further improve its structure and functional properties to suit some particular food system, which is of great significance for expanding the application of WPI in the food industry, and the effective use of whey. The chemical cross-linking has the characteristics of simple operation and rapid reaction, but some chemical cross-linkers could be toxic, such as formaldehyde and glutaraldehyde [6]. Therefore, it is becoming a hot spot to further explore safe and non-toxic chemical cross-linking methods.

Citric acid (CA) is inexpensive, non-toxic, and harmless, and is widely used in the food industry [7]. It has a unique carboxyl structure [8] and excellent cross-linking properties, so citric acid is gradually applied as a safe, efficient, and inexpensive cross-linking agent. Many studies have shown that citric acid can be used to cross-link proteins and improve their properties. Xu et al. [9] used citric acid to cross-link wheat-derived gliadin for 4 h at 50 °C or 75 °C, to unravel the mechanism of citric acid cross-linking. The free amino group of the protein undergoes a nucleophilic substitution reaction with a carboxyl group in citric acid to form a new amide bond, and citric acid is used to modify proteins by acylation during this process. Citric acid is a tricarboxylic acid, when more than one carboxyl group in the citric acid molecule participates in the reaction, protein inter- or intra-molecular cross-linking would occur [9,10]. Reddy et al. [10] used citric acid to cross-link soy protein, gliadin, and zein fibers, respectively, successfully increasing their breaking tenacity and breaking elongation. Bagheri et al. [11] also successfully prepared whey protein particles after the pretreatment of the transglutaminase-induced or citric acid-mediated cross-linking. Furthermore, Nourbakhsh et al. [12] further applied citric acid to the cross-linking of WPI hydrolyzed peptides to form embedding particles of gallic acid. Meanwhile, citric acid cross-linking significantly impacted the textural, functional and caffeine release behavior of WPI-caffeine gels [13]. Although many studies have successfully applied citric acid for cross-linking, there has been no literature about the changes in functional properties of WPI after cross-linking with citric acid. Therefore, the objective of this study was to investigate the effects of citric acid cross-linking modification on the functional properties of WPI, under different pH and citric acid concentrations. It is hoped that the functional properties of whey protein isolates will be improved by rapid, efficient, safe, and non-toxic chemical processes, providing a basic support for the food industry to obtain novel functional protein ingredients.

## 2. Results and Discussion

### 2.1. Surface Hydrophobicity

**Figure 1:**
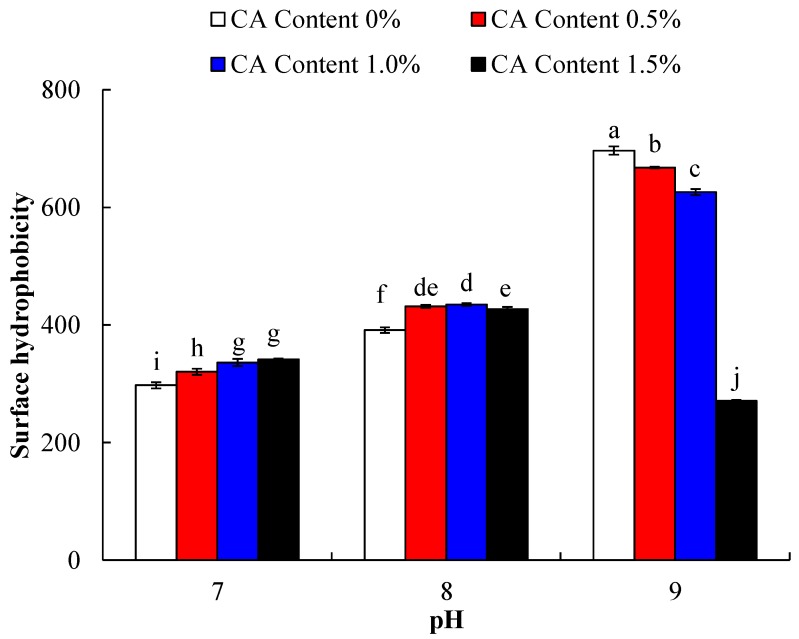
Surface hydrophobicity of WPI incubated with CA (0, 0.5, 1.0 and 1.5%) at pH 7.0, pH 8.0, or pH 9.0, at 50 °C for 6 h. Error bars represent the standard deviation of the mean of triplicate experiments. Values with different letters are significantly different (*p* < 0.05).

The change of surface hydrophobicity is related to the alteration of protein structure. Surface hydrophobicity is also a vital factor in emulsifying and foaming capacities. Surface hydrophobicity of WPI incubated with CA at pH 7.0, pH 8.0, or pH 9.0, is shown in Figure 1. Two-way analysis of variance (ANOVA) found that both main effects of CA content and pH, and their associated interaction, were significant (*p* < 0.05). Additionally, compared with untreated WPI, the surface hydrophobicity of CA-treated WPI gradually enhanced with the citric acid, increased from 0% to 1.5% at pH 7.0 and 8.0 (*p* < 0.05). It might be attributed to the fact that the hydrophobic groups buried in WPI were exposed during cross-linking with citric acid. Lakkis et al. [14] also found that acylation modification increased the surface hydrophobicity of BSA, casein, and WPC. Furthermore, it was also verified that the exposed hydrophobic groups during protein denaturation led to an increase of surface hydrophobicity [15]. However, there was a significant downward trend in surface hydrophobicity of WPI-CA conjugated with the citric acid increased at pH 9.0 (*p* < 0.05). For instance, surface hydrophobicity of WPI cross-linked with 1.5% citric acid at pH 9.0 showed a sharp decline. This was probably due to the formation of insoluble aggregates [16]. Moreover, the decrease in surface hydrophobicity of WPI-CA may be ascribed to the shielding effect of a large negative charge on proteins, which reduced ANS (8-Anilino-1-naphthalenesulfonic acid) binding to ligands [17], caused by the introduction of citric acid and the alkaline environment of pH 9.0. Meanwhile, WPI and WPI-CA conjugate had a larger surface hydrophobicity at higher alkaline conditions. The higher cross-linking degree would occur with the increasing of pH, potentially due to the more extensive deprotonation of amino groups, and thus more nucleophilic attacks. Furthermore, this may also be due to the unfolding of WPI which occurred significantly at pH 9.0, and its hydrophobic groups were exposed to the protein surface [18]. It was also reported that 1% WPI solutions had higher surface hydrophobicity at pH 9.0 than at pH 7.0 at 50 °C [18].

### 2.2. Zeta Potential

**Figure 2:**
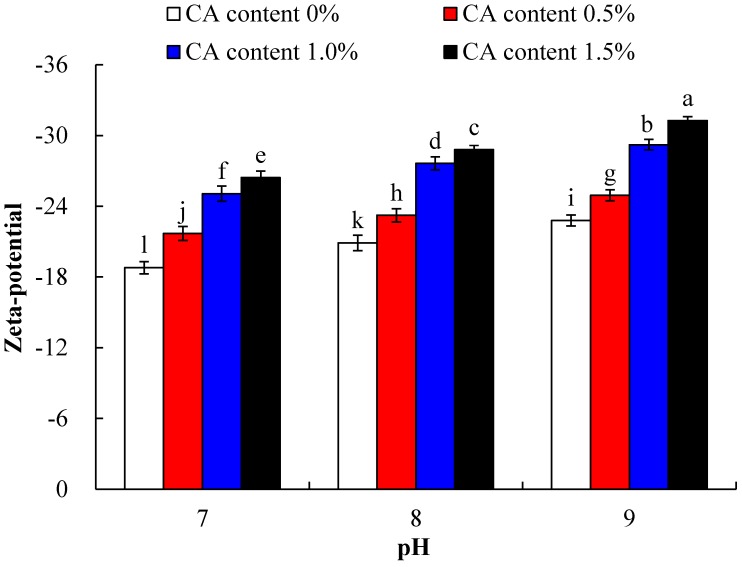
Zeta potential of WPI incubated with CA (0, 0.5, 1.0 and 1.5%) at pH 7.0, pH 8.0, or pH 9.0, and at 50 °C for 6 h. Error bars represent the standard deviation of the mean of triplicate experiments. Values with different letters are significantly different (*p* < 0.05).

Effects of pH and CA concentration on zeta potential of WPI and WPI-CA, were presented in Figure 2. Two-way ANOVA showed that both main effects of pH and CA content were significant, along with their associated interaction (*p* < 0.05). Overall, compared to WPI without CA, zeta potential of WPI-CA decreased markedly with the increase of pH and CA content. It was suggested that an increase of negative charges on the surface of protein occurred when the reaction degree increased, which could mean that cationic amino groups on proteins were converted to anionic residues, after WPI was modified by citrate acylation and cross-linking. Shilpashree et al. [17] investigated the effect of succinylation on milk protein concentrate and found that the zeta potential of acylation-treated milk protein concentrate after acylation decreased with the increasing degree of succinylation.

### 2.3. Protein Solubility

**Figure 3:**
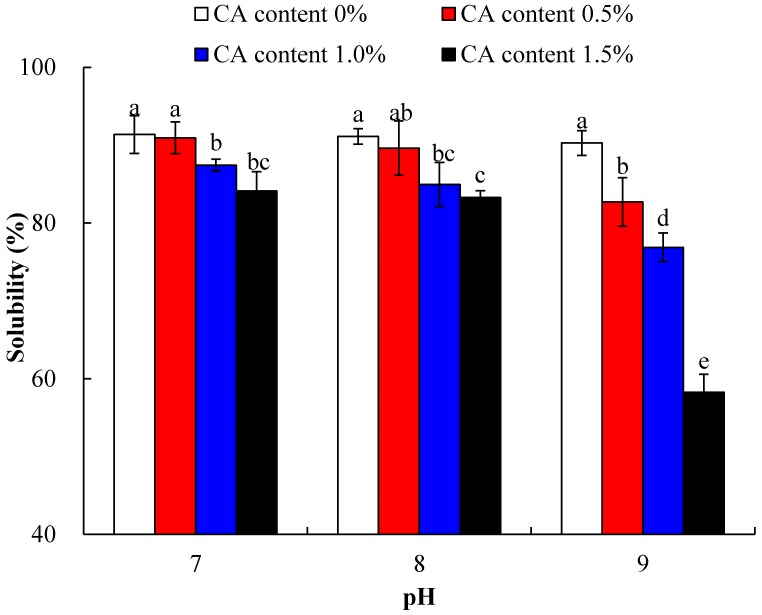
Solubility of WPI incubated with CA (0, 0.5, 1.0 and 1.5%) at pH 7.0, pH 8.0, or pH 9.0, and at 50 °C for 6 h. Error bars represent the standard deviation of the mean of triplicate experiments. Values with different letters are significantly different (*p* < 0.05).

In general, good solubility could affect its other functional properties in favorable manner [19]. Protein solubility of native and modified WPI responded to changes in pH, and the content of citric acid is indicated in Figure 3. Two-way ANOVA showed that both the two main effects of CA content and pH, and their interaction were significantly effective (*p* < 0.05). When citric acid content increased from 0% to 1.5%, the protein solubility gradually decreased (*p* < 0.05), regardless of pH 7.0, 8.0, or 9.0, which decreased from 91% to 84%, 91% to 83%, and 90% to 58%, respectively. It may be due to the fact that as the pH increased, the amino group underwent more extensive deprotonation under alkaline conditions, resulting in more nucleophilic substitution reactions, which favored the greater degree of cross-linking [9]. Larger molecular weight cross-linked products result in a gradual decrease in solubility. In general, reduction of solubility was expected with the increase in hydrophobicity, as the latter rendered the molecules less water-soluble, and perhaps forced neighboring macromolecules to aggregate with their hydrophobic parts. It is worth mentioning that when the citric acid content was 1.5% at pH 9.0, the solubility of WPI-CA was minimized. At this time, some insoluble polymer was produced. Therefore, the cross-linking modification of citric acid had a significant effect on the solubility of WPI, and may have produced some large molecular size proteins with poor solubility, resulting in the solubility decrease of WPI-CA.

### 2.4. Emulsifying Properties

**Figure 4:**
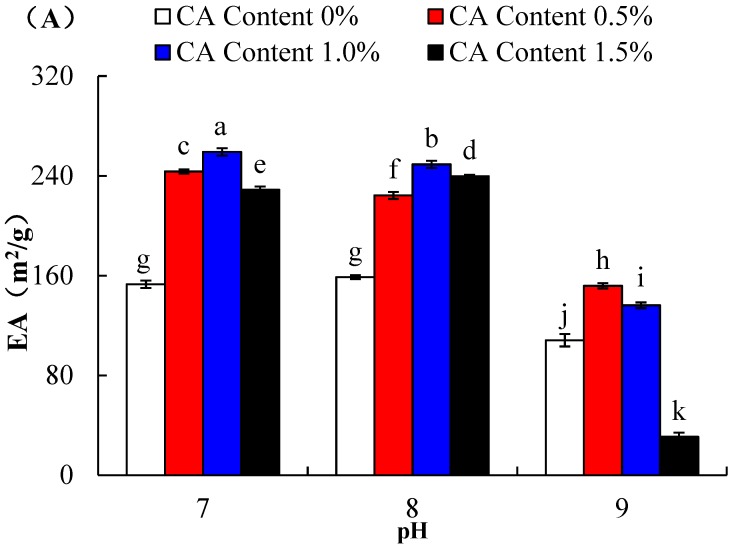

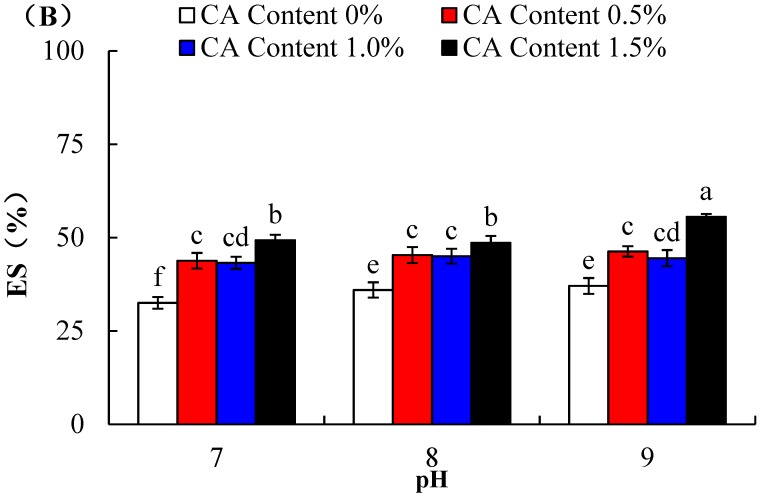
Emulsifying activity (**A**) and emulsion stability (**B**) of WPI incubated with CA (0%, 0.5%, 1.0%, and 1.5%) at pH 7.0, pH 8.0, or pH 9.0, and at 50 °C for 6 h. Error bars represent the standard deviation of the mean of triplicate experiments. Values with different letters are significantly different (*p* < 0.05).

Emulsifying activity of WPI incubated with CA (0, 0.5, 1.0 and 1.5%) at pH 7.0, pH 8.0, and pH 9.0, is shown in Figure 4A. Two-way ANOVA presented that the main effects of pH and CA content were significant, and also their associated interaction (*p* < 0.05). Compared with untreated WPI, WPI-CA had higher emulsifying activity. With the increase of citric acid content from 0% to 1.5%, the emulsifying activity of WPI increased significantly, and afterward decreased at the same pH value (*p* < 0.05). In general, surface hydrophobicity and solubility of acylated proteins may play a decisive role in improving emulsifying properties [20]. In this study, when the citric acid increased from 0% to 1.0%, surface hydrophobicity of WPI-CA improved, as shown in Figure 1, accounting for the enhancement of an increase in emulsifying activity of WPI-CA at pH 7.0 and 8.0, respectively. Additionally, at the oil-water interface, the protein located lipophilic residues in the oil phase and hydrophilic residues in the water phase, thus reducing the surface tension at the interface. Acylation increased lipophilic and hydrophilic residues exposure of proteins, and improved emulsifying activity [21]. However, with the cross-linking degree increased, the solubility of the WPI-CA gradually decreased, adversely impacting its emulsifying activity. Specifically, there was the lowest emulsifying activity of WPI at the citric acid level of 1.5% and pH 9.0, possibly due to its lowest solubility induced from the production of higher molecular size proteins during the cross-linking of WPI and CA. Meanwhile, the decrease in EA was also due to the sharp increase of net negative charges of high level acylation [22].

Emulsion stability (ES) of WPI incubated with CA (0, 0.5, 1.0 and 1.5%) at pH 7.0, pH 8.0, or pH 9.0, is shown in Figure 4B. An analysis of variance (two-way ANOVA) found that only the main effects of pH and CA content were significant (*p* < 0.05), whilst interaction between two factors was not significant (*p* > 0.05). Overall, ES of WPI-CA increased significantly at pH 7.0, 8.0, and 9.0, when the citric acid increased from 0% to 1.5%. After the cross-linking of WPI and CA, the cationic amino groups on proteins were converted to anionic residues, resulting in an increase in the net negative charge. This benefited the formation of charged layers of proteins around fat globules, leading to mutual repulsion, decreasing interfacial energy and retarded aggregation [21], favoring the formation of the stable emulsion of WPI-CA. Meanwhile, the increase in pH slightly promoted the ES of WPI-CA conjugate. Lawal et al. [23] reported a similar result, where succinylated protein showed improved ES over the native protein, in the pH range of 4.0 to 10.0.

### 2.5. Foaming Properties

**Figure 5:**
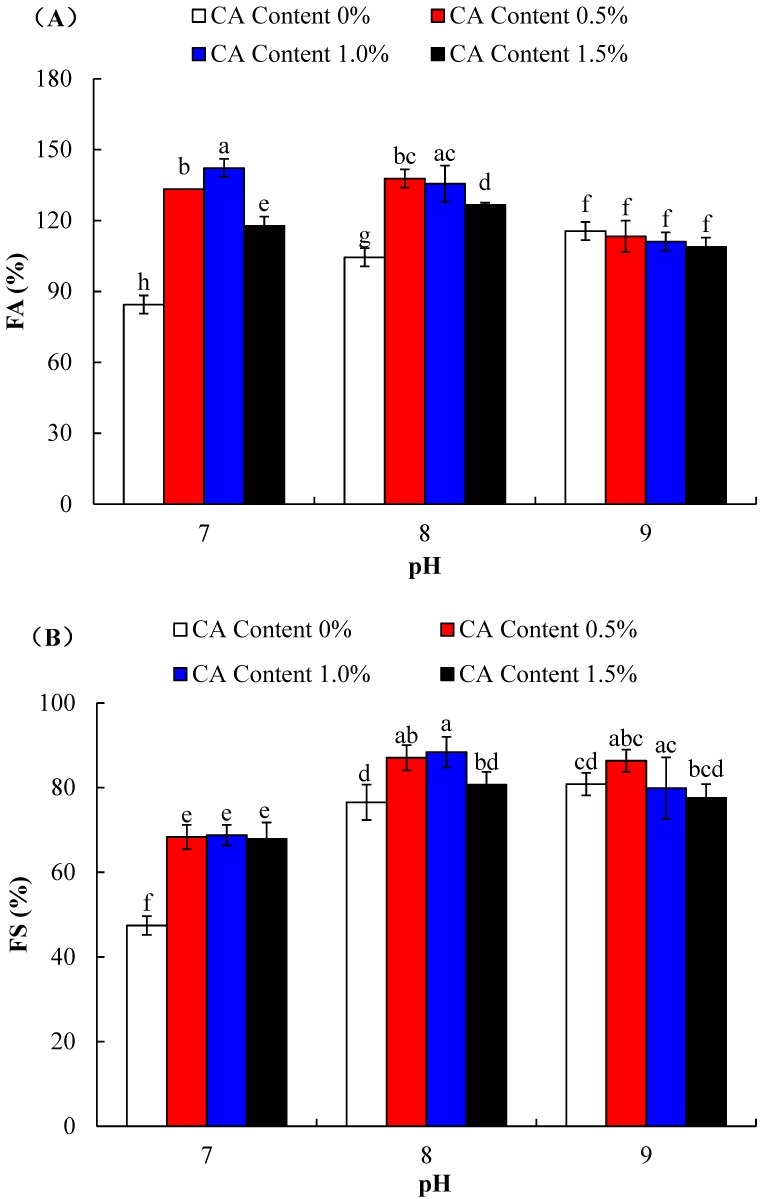
Foaming ability (**A**) and foam stability (**B**) of WPI incubated with CA (0%, 0.5%, 1.0% and 1.5%) at pH 7.0, pH 8.0, or pH 9.0, and at 50 °C for 6 h. Error bars represent the standard deviation of the mean of triplicate experiments. Values with different letters are significantly different (*p* < 0.05).

Foaming ability (FA) and foaming stability (FS) of WPI-CA at pH 7.0, pH 8.0, or pH 9.0, is shown in Figure 5A,B. Two-way ANOVA showed that the main effects of pH and CA content and their interaction, were significant on foaming properties (*p* < 0.05). Overall, from Figure 5A, compared with untreated WPI, WPI-CA had higher foaming ability at pH 7.0 and 8.0 (*p* < 0.05). The improvement in foaming ability of WPI-CA could be due to the exposure of the hydrophobic region, enhancing the rate of adsorption of the protein to the air-water interface. There was a decrease of FA when citric acid increased to 1.5%. Furthermore, the addition of citric acid had no a significant effect on the foam activity of WPI at pH 9.0. Citric acid-mediated cross-linking of proteins decreased inter-protein interactions [7]. Excessive modification of citric acid could result in fewer protein–protein interactions, and the insoluble portion of the protein did not contribute to foam formation, being unfavorable to the improvement in foaming. Moreover, protein aggregation formed by the cross-linking of WPI and CA could impede the improvement of FA. Meanwhile, the foam activity of WPI without citric acid was positively correlated with the pH value. Similar results were obtained by Malgorzata et al. [24], who found that the foam activity of whey protein was higher at pH 9.0, compared to pH 7.0. This was due to the protein structure unfolding under an alkali environment, which led to the exposure of some functional groups and the increase of molecular flexibility, which was helpful for the foam activity of WPI.

From Figure 5B, compared with untreated WPI, WPI-CA had higher foaming stability at pH 7.0 and 8.0 (*p* < 0.05). In general, the foaming properties of protein and its ability to form film at the air-water interface are relevant [25], and the aggregates could result in the best foam stability, probably due to network formation within the film [26]. Therefore, with the gradual occurrence of the cross-linking between WPI and CA, the products could have better foaming stability. Additionally, the cross-linking of WPI and CA increased the surface hydrophobicity, and the exposed hydrophobic groups of WPI-CA conjugate stabilized the interface between air and water in foam formation, thus the increase of surface hydrophobicity could contribute to the improvement of foam stability. Shilpashree et al. [17] also verified that the foaming capacity and stability of milk protein concentrate were improved after its succinylation. However, excessive cross-linking led to the WPI-CA with poor solubility, as shown in Figure 2, thereby reducing the amount of whey protein that could be used to form a film, and resulting in the inability of WPI to form an elastic film at the air-water interface. This could be used to explain the decrease in foaming stability of WPI-CA at pH 8.0 with 1.5% citric acid, or the insignificant difference in foaming stability of WPI-CA at pH 9.0.

## 3. Materials and Methods

### 3.1. Materials

Whey protein isolate with 93.5% protein content was purchased from Mullins Whey Inc., Mosinee, WI, USA. Citric acid (≥99.5%) was obtained from Macklin, Shanghai, China. Sodium dodecyl sulfates (SDS), 8-Anilino-1-naphthalenesulfonic acid (ANS) were purchased from Sigma-Aldrich Co. (St. Louis, MO, USA). All other chemicals used were of analytical grade.

### 3.2. Citric Acid-Mediated Cross-Linking of WPI

WPI solutions (5% *w*/*v*) were prepared by dissolving WPI powders in deionized water and stirring at room temperature for 2 h. Sodium azide (50 mg/L) was added as an antimicrobial agent. The solutions were stored at 4 °C overnight to ensure complete dissolution. Citric acid was added to the WPI solutions with a final concentration of 0.5%, 1.0%, and 1.5%. The WPI solutions without citric acid were served as a control. Then, all samples were divided into three portions, and the pH values were adjusted to 7.0, 8.0, and 9.0, using NaOH solution (20 mol/L). All sample solutions were treated at 50 °C for 6 h. After cross-linking, the samples were lyophilized and stored.

### 3.3. Surface Hydrophobicity

The surface hydrophobicity was determined using 1-anilinonaphthalene-8-sulphonate (ANS) as a fluorescent probe, according to the method of Kato et al. [15]. The samples were diluted with 0.01 mol/L sodium phosphate buffer at pH 7.0, and the concentration was from 1 to 5 mg/mL. Twenty microliters of 8.0 mmol/L ANS was mixed with 4 mL of samples. The mixture was shaken and kept in the dark for 15 min to react completely. The relative fluorescence intensity of samples with ANS was measured at room temperature using a Hitachi F4500 fluorescence spectrometer (Hitachi, Tokyo, Japan). Excitation and emission wavelength were set at 390 and 470 nm, respectively, with slit widths of 5 nm. The initial slope of fluorescence intensity with increasing protein concentration was calculated as the surface hydrophobicity of the samples.

### 3.4. Zeta Potential

The zeta-potential values of the samples were measured using a Zetasizer Nano-ZS90 (Malvern Instruments Ltd., Worcestershire, UK). Before the detection, the protein concentrations were adjusted to 2 mg/mL with 0.01 mol/L sodium phosphate buffer (pH 7.0), and the measurements were carried out at 25 °C.

### 3.5. Solubility

The solubility of proteins was determined according to the modified method of Smith et al. [27]. Sample powder was dispersed (1% *w*/*v*) in 0.01 mol/L sodium phosphate buffer (pH 7.0). The protein dispersions were centrifuged at 10,000 rpm, and at 20 °C for 25 min. The protein concentrations of samples were respectively measured in the non-centrifuged dispersions and in the supernatants after centrifugation using the bicinchoninic acid (BCA) method, at 562 nm using the microplate reader. The solubility of the sample was calculated using the following equation:

(1) $$\mathrm{Solubility}\left(\%\right)=\frac{C_{1}}{C_{2}}\times 100\%$$

where C_1_ is the protein concentration of the supernatants (mg/mL), and C_2_ is the protein concentration of the initial dispersions (mg/mL).

### 3.6. Emulsifying Activity and Emulsion Stability

The emulsifying properties of the protein were determined by the turbidity method [28]. Furthermore, 1 mL of soybean oil and 3 mL of sample (0.5 mg/mL) were added together, and the mixture was homogenized for 2 min at 10,000 r/min. After the homogenization, 25 μL of emulsion samples were placed for 0 min and 10 min, and immediately mixed with 5 mL of 0.1% SDS solution. The absorbance of the emulsion was read at 500 nm, using 0.1% SDS solution as a reference. Emulsifying activity (EA) and emulsion stability (ES) were calculated using the following formulas:

(2) $$\mathrm{EA}\;\left(m^{2}/g\right)=\frac{2\times 2.303\times D\times A}{l\times \left(1-\emptyset \right)\times C\times 10}$$

(3) $$\mathrm{ES}\;\left(\%\right)=\frac{A_{10}}{A}\times 100\%$$

where D is the dilution multiple, ∅ is the volume fraction of the oil phase, A and A_10_ are the absorbance at 500 nm after 0 min and 10 min, respectively, C is the volume fraction of protein in the emulsion, and l is the path length of cuvette (1 cm).

### 3.7. Foaming Activity and Foam Stability

Foaming activity (FA) and foaming stability (FS) were determined according to the method described by He et al. [29], with slight modifications. Twenty milliliters of sample solution (50 mg/mL) were stirred at a rate of 10,000 r/min for 2 min using a high-speed mixer at room temperature, and the resulting sample was transferred to a graduated cylinder. The volume of the mixture was recorded after whipping. The sample was placed for 30 min, and its volume was recorded again. FA and FS were calculated using the following formulas:

(4) $$\mathrm{FA}\;\left(\%\right)=\frac{V_{2}-V_{1}}{V_{1}}\times 100\%$$

(5) $$\mathrm{FS}\;\left(\%\right)=\frac{V_{3}}{V_{2}}\times 100\%$$

where V_1_ is the volume of the initial sample solution, V_2_ is the volume of the mixture at 0 min after whipping, and V_3_ is the volume of the mixture left for 30 min after whipping.

### 3.8. Statistical Analysis

In these tests, all experiments were repeated at least three times. The data were analyzed by performing analysis of variance (one-way and two-way ANOVA) and the Duncan’s test using SPSS software 22.0 (SPSS Inc., Chicago, IL, USA). Confidence level was set for *p* < 0.05 level.

## 4. Conclusions

WPI-CA conjugates were prepared at a citric acid concentration (of 0%, 0.5%, 1.0% and 1.5%), under neutral or alkali conditions, at 50 °C for 6 h. Results of surface hydrophobicity stated that WPI underwent structural modification with the occurrence of cross-linking. The citric acid cross-linking also induced a slight reduction in solubility of WPI-CA, but improved the emulsifying and foaming properties of WPI. In addition, zeta potential of WPI-CA decreased remarkably with the increase of pH and CA content. Therefore, citric acid-mediated cross-linking under non-acidic conditions could lead to structural modification and enhance emulsifying and foaming properties of WPI. Furthermore, according to the improvement of WPI-CA on functional properties, the modified WPI could be used in food systems where emulsifying or foaming plays a critical role.
